# Action of the Heart

**Published:** 1858-07

**Authors:** 


*Action of the Heart.*—Dr. Pavy, in his lectures on Physiology, at Guy's Hospital, has made some assertions, based upon experiments on dogs and on examinations of the action of the heart in a man with congenital pressure of the sternum, which run counter to the commonly received opinions of physiologists. The common teaching is stated by Dr. P. in these words:

"The corresponding cavities of the two sides act simultaneously, while the auricles and ventricles respectively contract and dilate alternately. The ventricles are continuously engaged in contracting and dilating, and occupy an equal period of time in each. The auricles, however, not only dilate during the whole of the ventricular contraction, but continue dilating during the first stage of the ventricular diastole, and do not contract till its second stage, thus leaving a period in the heart's action when all its cavities alike are undergoing dilatation."

This description the lecturer considers perfectly good for reptiles, but altogether incorrect as an expression of the action of the heart in man, and the mammalia generally. According to him, this is what takes place in them:

The contraction of the ventricles is sharp and rapid, and does not appear to occupy nearly so much as half the period of the heart's action. The ventricular contraction communicates a sudden impulse to the auricles, occasioning in them a distinct pulsation, which is instantly followed by a peculiar thrill, wave, or vermicular movement, running through the auricular parietes down to the ventricle. This thrill or wave is coincident with the passage of the blood from the auricle into the ventricle, and takes place so instantaneously after the ventricular contraction, that the one movement appears to run or continue itself into the other. There is then a pause, which seems comparatively of considerable duration, and which is succeeded by a recommencement of the heart's action, beginning with the ventricular contraction.

